# Identification and Verification of Tumor Immune Microenvironment-Related Prognostic Genes in Kidney Renal Clear Cell Carcinoma

**DOI:** 10.1155/2022/5563668

**Published:** 2022-01-27

**Authors:** Gangcheng Kong, Yixuan Wang, Yuanjian Huang, Zhen Shi

**Affiliations:** ^1^Department of Hepatobiliary and Pancreatic Surgery, The First Affiliated Hospital, School of Medicine, Zhejiang University, Hangzhou, Zhejiang, China; ^2^Cardiovascular Surgery, Wuhan Union Hospital, Huazhong University of Science and Technology, Wuhan, Hubei, China; ^3^Department of Colorectal Surgery, The First Affiliated Hospital of Nanjing Medical University, Nanjing, Jiangsu, China; ^4^Department of Plastic Surgery, The Second Affiliated Hospital Zhejiang University School of Medicine, Hangzhou, Zhejiang, China

## Abstract

**Background:**

The tumor immune microenvironment is vital to kidney renal clear cell carcinoma (KIRC) progression, and immunotherapies have been shown to be effective in the management of KIRC. However, the prognostic genes associated with the tumor immune microenvironment in KIRC have not been fully identified. We obtained the KIRC RNA sequencing data and the clinical characteristics from The Cancer Genome Atlas (TCGA) and the International Cancer Genome Consortium (ICGC) database. We screened the gene modules associated with the tumor immune microenvironment based on the ESTIMATE algorithm and weighted gene coexpression network analysis (WGCNA). Univariate Cox analysis and the LASSO method were used to construct a prognostic model. Receiver Operating Characteristic (ROC) curve analysis was performed to evaluate the accuracy of our risk model. TIMER and Single-Sample Gene Set Enrichment Analysis (ssGSEA) were used to explore the correlation between prognostic genes and immune cell infiltration.

**Results:**

Fifty-four genes in modules were significantly associated with the overall survival (OS) time of patients with KIRC. Furthermore, 12 hub genes were selected to construct the prognostic model. The prognostic model showed superior accuracy in both TCGA and ICGC cohorts using ROC curve analysis. Systematic analysis of immune cell infiltration revealed that nine genes were significantly correlated with levels of tumor-infiltrating immune cells.

**Conclusions:**

Our findings indicated that the tumor immune microenvironment was an important determinant of KIRC outcomes and revealed potential biomarkers for predicting patient OS and for targeted immunotherapies.

## 1. Introduction

Malignant kidney tumors are common worldwide, accounting for 2–3% of all cancers [1]. Based on different molecular signatures, there are various histological subtypes of kidney cancer. Clear cell renal cell carcinoma (ccRCC) is the most common subtype and accounts for 70–80% of all kidney cancer cases [2, 3]. Etiological factors, including genetic factors and lifestyle variables, such as smoking, obesity, and hypertension, participate in kidney tumorigenesis. At the gene level, kidney renal clear cell carcinoma (KIRC) is associated with the loss of chromosome 3p, and mutations in *VHL*, *PBRM1*, *SETD2*, and *BAP*1 are involved in KIRC progression and metastases [2]. Patients with advanced ccRCC have a poor prognosis. Anatomical, histological, clinical, and molecular factors influence patient outcomes. Despite advances in diagnosis, surgery, and drug treatment, the clinical outcomes of patients with KIRC are still unsatisfactory.

The tumor microenvironment (TME) is involved in tumor progression. Various cells, including fibroblasts, endothelial cells, and immune cells, and extracellular components surrounding tumor cells play vital roles in cancer biology [4]. Tumor-infiltrating immune cells have been widely studied and are known to target tumor cells and inhibit tumor growth, exhibiting antitumor activity. In contrast, these cells can also exhibit protumor activity and promote tumor development and metastases [5, 6]. For example, regulatory T cells modulate the functions of effector T cells and suppress their proliferation. Tumor-associated myeloid cells, such as tumor-associated macrophages (TAMs) and myeloid-derived suppressor cells (MDSCs), are important tumor-infiltrating immune cells. In general, the high frequency of TAMs is associated with poor prognosis in human cancers [7]. Compared with other solid tumors, ccRCC has a unique immune microenvironment. Infiltrating CD8^+^ T cells in ccRCC tumors are relatively abundant but show impaired tumor killing ability, and patients with increased levels of CD8^+^ T cells in tumors usually have poor outcomes [8]. Recently, immune checkpoint inhibition has been shown to be an effective method in the treatment of kidney cancer. Thus, investigation of the TME in ccRCC and elucidation of the underlying mechanisms are important for improvement of the diagnosis and treatment of ccRCC. Many computational methods, including the Estimate of Stromal and Immune cells in Malignant Tumors using Expression data (ESTIMATE) algorithm and Tumor Immune Estimation Resource (TIMER), can help improve our understanding of the roles of the TME during ccRCC tumorigenesis and progression [9, 10]. In the current study, we used weight gene coexpression network analysis (WGCNA) to identify KIRC immune-related gene modules and constructed a prognostic model based on least absolute shrinkage and selection operator (LASSO) Cox regression analysis. LASSO is a variable selection method to shrink and select variates for regression. Variable selection methods assume that the “signals” are sparse, while dimension reduction methods assume that all covariates carry some signals. In genetic data analyses of pan cancers in TCGA data, LASSO is pervasively adopted to be applied in genetic data for the univariate Cox regression analysis.

Twelve genes in our risk model significantly influenced patient survival. Our results provided insights into the mechanisms through which the TME affects clinical outcomes in patients with KIRC and identified potential prognostic and therapeutic targets for KIRC.

## 2. Materials and Methods

### 2.1. Datasets

RNA sequencing data containing RSEM normalized data and the clinical characteristics of KIRC patients (537 primary tumor samples, 537 patients) were obtained from TCGA database. Cases with incomplete clinical data, overall survival time less than 30 days, and obvious outlier RNA sequence data were removed. Finally, the data from 499 patients were analyzed in this study. The stromal and immune scores of TCGA KIRC dataset were calculated using the “estimate” package in R language. In the current study, TCGA cohort was separated randomly at a 2 : 1 ratio; 349 samples were used to generate the prognostic model, and 150 samples were used for validation. Another cohort of KIRC patient with RNA sequencing data was obtained from the ICGC database. Cases with incomplete clinical data and obvious outlier RNA sequence data were removed. Finally, 70 patients with RNA sequencing data and clinical characteristics were included in this study as ICGC validation dataset. The clinical information for patients from TCGA and ICGC is shown in Table 1. The gene expression profile (GSE28490) based on the platform of GPL570 (Affymetrix Human Genome U133 Plus 2.0 Array) was downloaded from the GEO database (http://www.ncbi.nlm.nih.gov/geo/). The dataset GSE28490 contains the gene expression information of human immune cell subset. The raw data were normalized using the robust multi-array average (RMA) algorithm through the Affy package of Bioconductor (http://www.bioconductor.org/).

### 2.2. WGCNA to Identify Key Modules

WGCNA was performed using the “WGCNA” package in R language [11]. In total, 499 RSEM normalized RNA sequencing data from TCGA KIRC cohort were included for WGCNA analysis. Genes that were undetectable in more than 50 samples were filtered out; then, we chose the top 5000 genes with the high deviation to construct a network. The unsigned coexpression networks were established based on the best soft thresholding power *β*. Then we calculated the coexpression similarity and transformed the similarity matrix to the weighted adjacency matrix. Next, we transformed the weight adjacency matrix into a topological overlap matrix (TOM) to detect gene connectivity in the network. Finally, based on the TOM, gene dendrograms over 30 were produced to construct coexpression gene modules. We merged similar modules based on a height cut of 0.25 and calculated the module membership and gene significance to evaluate the gene relationships between tumor stage, tumor grade, immune score, and estimate score. Genes in modules that were closely related to these four traits were selected for further analysis.

### 2.3. Functional Annotation Analysis

Functional annotation of Gene Ontology (GO) terms and Kyoto Encyclopedia of Genes and Genomes (KEGG) pathways for the genes in the most related modules of the WGCNA were performed using the “clusterProfiler” R package [12]. The *P* value was adjusted by the Benjamini and Hochberg method [13].

### 2.4. Construction of a KIRC Prognostic Model

Univariate Cox analysis and the LASSO method were used to identify genes that significantly influence patient survival [14]. First, we used univariate Cox regression analysis to find the prognostic genes in green/yellow and tan modules. Then, genes that significantly influenced patient clinical outcomes (*P* < 0.001) were used for LASSO analysis. TCGA discovery dataset with 349 patient data was used to construct the prognostic model.

### 2.5. Survival Analysis

Survival analysis was used to investigate the relationships between different gene expression levels and patient survival. According to the gene expression level, patients with KIRC were divided into the high-expression group and low-expression group. Based on the risk score, patients were divided into the high-risk group and low risk group. Kaplan-Meier survival analysis was performed using “survival” and “survminer” R language packages.

### 2.6. Evaluation of the KIRC Prognostic Model

The accuracy of the KIRC prognostic model generated by LASSO Cox regression analysis was assessed by using the Receiver Operating Characteristic (ROC) curve method. The sensitivity and specificity of our prognostic model for 1-, 3-, and 5-year overall survival (OS) in TCGA KIRC discovery and test cohorts were evaluated using the “survivalROC” R package [15]. ICGC cohort was used as the external evaluation dataset.

### 2.7. Analysis of Immune Cell Infiltration

TIMER is a comprehensive resource for systematic analysis of immune cell infiltration across different cancer types (https://cistrome.shinyapps.io/timer/) [9]. We used TIMER to investigate the infiltration of immune cells, including B cells, CD8^+^ T cells, CD4^+^ T cells, macrophages, neutrophils, and dendritic cells, in TCGA KIRC patients. We analyzed the correlations between hub genes and immune cells in tumor tissues.

### 2.8. Single-Sample Gene Set Enrichment Analysis (ssGSEA)

KIRC RSEM normalized RNA-seq data were compared with the gene set using the “GSVA” package in R. The gene sets included 782 genes for predicting the proportions of 28 tumor-infiltrating immune cells in tumor tissues [16]. The features of cells exerting antitumor reactivity (including activated CD4^+^ T cells, activated CD8^+^ T cells, central memory CD4^+^ T cells, central memory CD8^+^ T cells, effector memory CD4^+^ T cells, effector memory CD8^+^ T cells, Th1 cells, Th17 cells, activated dendritic cells, natural killer NK T cells, and CD56 bright NK cells) and cells exerting protumor reactivity (including regulatory T cells, Th2 cells, immature dendritic cells, macrophages, MDSCs, neutrophils, plasmacytoid dendritic cells, and CD56dim NK cells) were obtained from recent publications [17, 18]. The ssGSEA score was normalized to the unity distribution. Scores for antitumor immunity and protumor suppression for each sample were calculated. The scores were plotted, and the correlation between antitumor immunity and protumor suppression in KIRC was analyzed by Pearson's correlation analysis.

## 3. Results

### 3.1. Immune Scores Were Closely Associated with KIRC Progression and Survival

**Figure 1:**
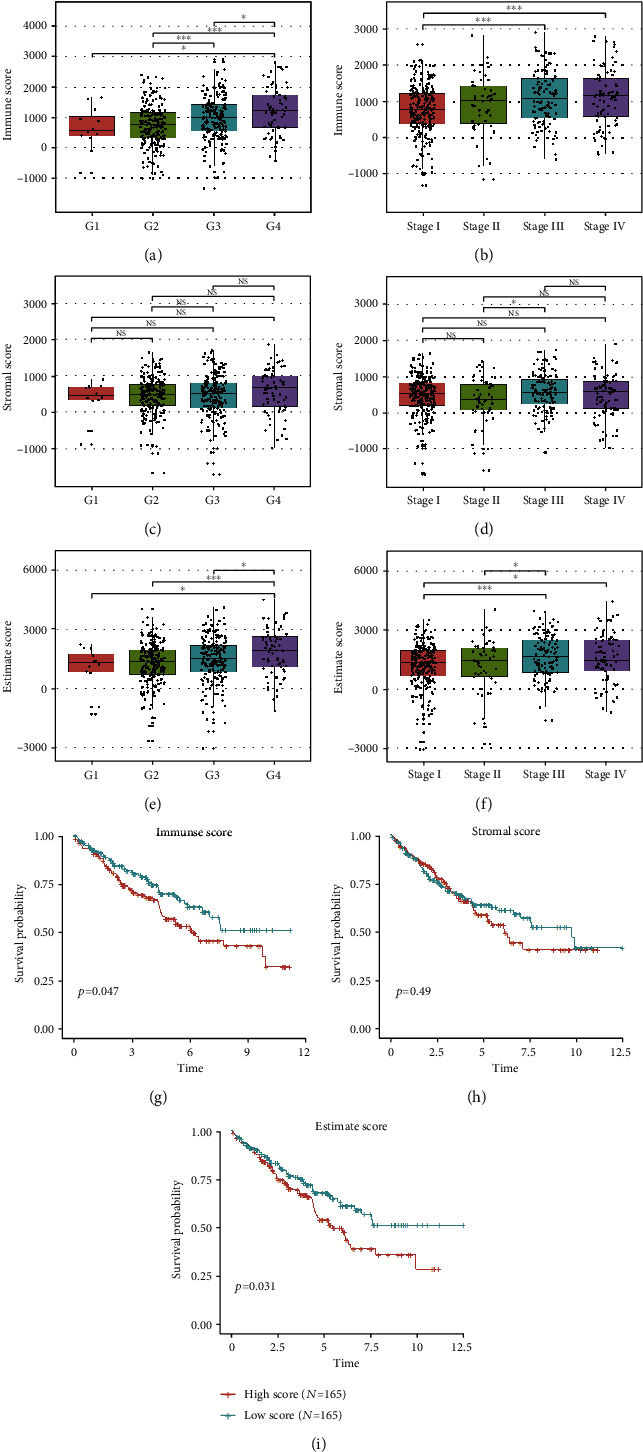
Immune scores and estimate scores were correlated with KIRC grade, stage, and outcome. (a–f) Immune scores and estimate scores were positively associated with grade and stage in KIRC, and stromal scores were comparable during KIRC progression (*t*-test; ns: not significant; ^∗^*P* < 0.05,  ^∗∗^*P* < 0.01, and^∗∗∗^*P* < 0.001). (g–i) Immune scores and estimate scores were significantly correlated with prognosis in patients with KIRC, whereas stromal scores were not significantly associated (log-rank test).

The stromal score, immune score, and estimate score reflect the infiltration levels of stromal cells and immune cells in tumor. In total, 499 patients with RNA sequencing data were included in this study, and the scores were calculated. First, we compared the difference of immune scores, stromal scores, and estimate scores between different stages of KIRC. Immune scores and estimate scores were significantly associated with KIRC progression, whereas stromal scores were comparable (Figures 1(a)–1(f)). To investigate the relationships between scores and patient prognosis, KIRC patients from TCGA cohort were divided into the high-score group (score > 67 percentile) and low-score group (score < 33 percentile). The results revealed that immune score and estimate score were significantly related to the OS of patients with KIRC. High immune score and high estimates indicated the poor prognosis (Figures 1(g) and 1(i)). However, the stromal score did not significantly affect clinical outcomes (Figure 1(h)). This observation highlighted the importance of the immune microenvironment in KIRC survival.

### 3.2. Screening Immune-Related Prognostic Gene Modules in KIRC Patients via WGCNA

**Figure 2:**
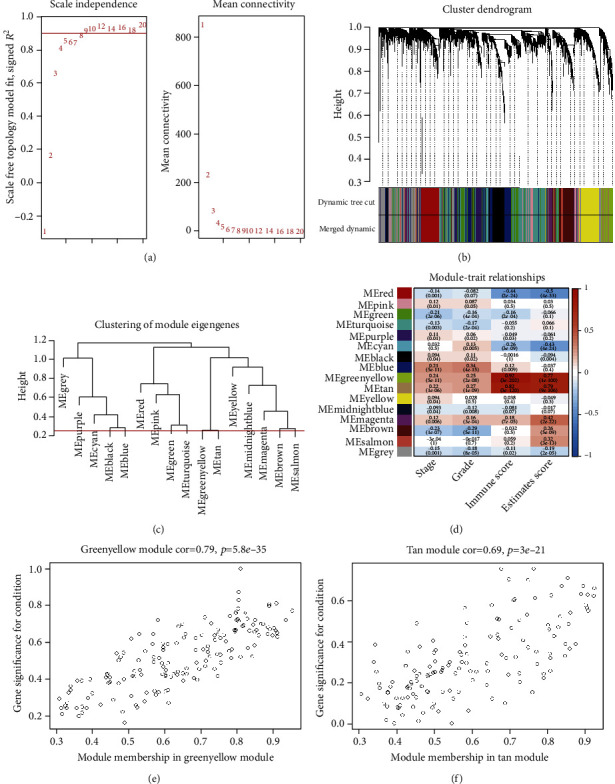
WGCNA analysis of KIRC data. (a) Analysis of scale-free index for various soft-threshold powers and mean connectivity for various soft thresholding powers. (b) Dendrogram of top 5000 high deviation genes clustered based on the TOM. Each branch represents a single gene; each color indicates a single module that contains weighted coexpressed genes. (c) Clustering of 16 module eigengenes. The merging threshold was shown as the red line. (d) Heatmap of the correlations between gene modules and stage, grade, immune score, and estimate score. ME green/yellow and ME tan modules were chosen for further analysis. (e, f) Gene correlation scatter plots for the green/yellow and tan module.

WGCNA is a method to identify key gene modules closely associated with clinical traits and scores generated by the ESTIMATE algorithm. Clinical and RSEM-normalized RNA sequencing data from 499 KIRC samples were used to construct a gene coexpression network. The best soft thresholding power *β* = 5 was calculated (Figure 2(a)) and used to calculate the adjacencies. 16 gene modules with sizes ranging from 46 to 753 genes were screened out based on TOM and dynamic tree clipping (Figure 2(b)). Similar modules were merged based on the height cut of 0.25 (Figure 2(c)). Finally, we determined 16 gene modules correlations with tumor stage, grade, immune score, and estimate score (Figure 2(d)). We assigned an arbitrary color for each coexpression module (red, pink, green, turquoise, purple, cyan, black, blue, green/yellow, tan, yellow, midnight blue, magenta, brown, salmon, and gray). These modules contained 374, 196, 394, 753, 159, 105, 314, 661, 158, 141, 407, 46, 173, 412, 108, and 599 genes, respectively. The non-co-expressed group was designated as gray. We calculated the module membership and gene significance of each module and chose the genes in green/yellow and tan modules, which were significantly associated with those four traits, for further analysis (Figures 2(e) and 2(f)).

### 3.3. Functional Enrichment Analysis of Highly Correlated Module Genes

**Figure 3:**
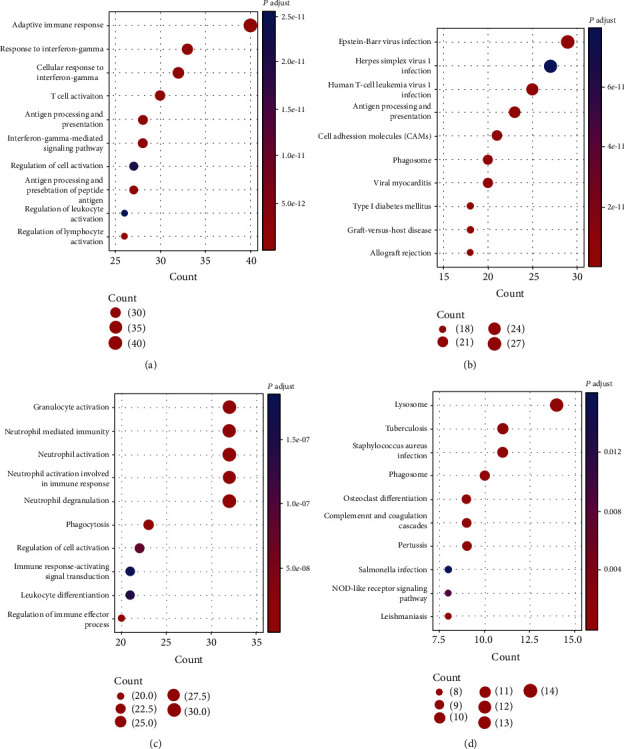
Functional enrichment analysis of genes in the green/yellow and tan modules in the KIRC patients. (a, b) Biological process analysis of GO enrichment and KEGG pathway analysis for genes in the green/yellow module. (c, d) Biological process analysis of GO enrichment and KEGG pathway analyses for genes in tan module.

There were 158 genes in the green/yellow module and 141 genes in the tan module. To further elucidate the functions of these genes, we performed GO and KEGG analyses. For genes in the green/yellow module, GO analysis showed that the top terms of biological processes (BPs) included adaptive immune response, response to interferon-gamma, and T-cell activation (Figure 3(a)). And these genes were enriched in immune-related pathways, including antigen processing and presentation and cell adhesion molecules (Figure 3(b)). For genes in the tan module, the top terms of BPs included granulocyte activation, neutrophil mediated immunity, and neutrophil degranulation (Figure 3(c)). And these genes were involved in innate immune responses, including lysosome, phagosome, and NOD-like receptor signaling pathways (Figure 3(d)).

### 3.4. Construction and Validation of a Prognostic Risk Model for KIRC

**Figure 4:**
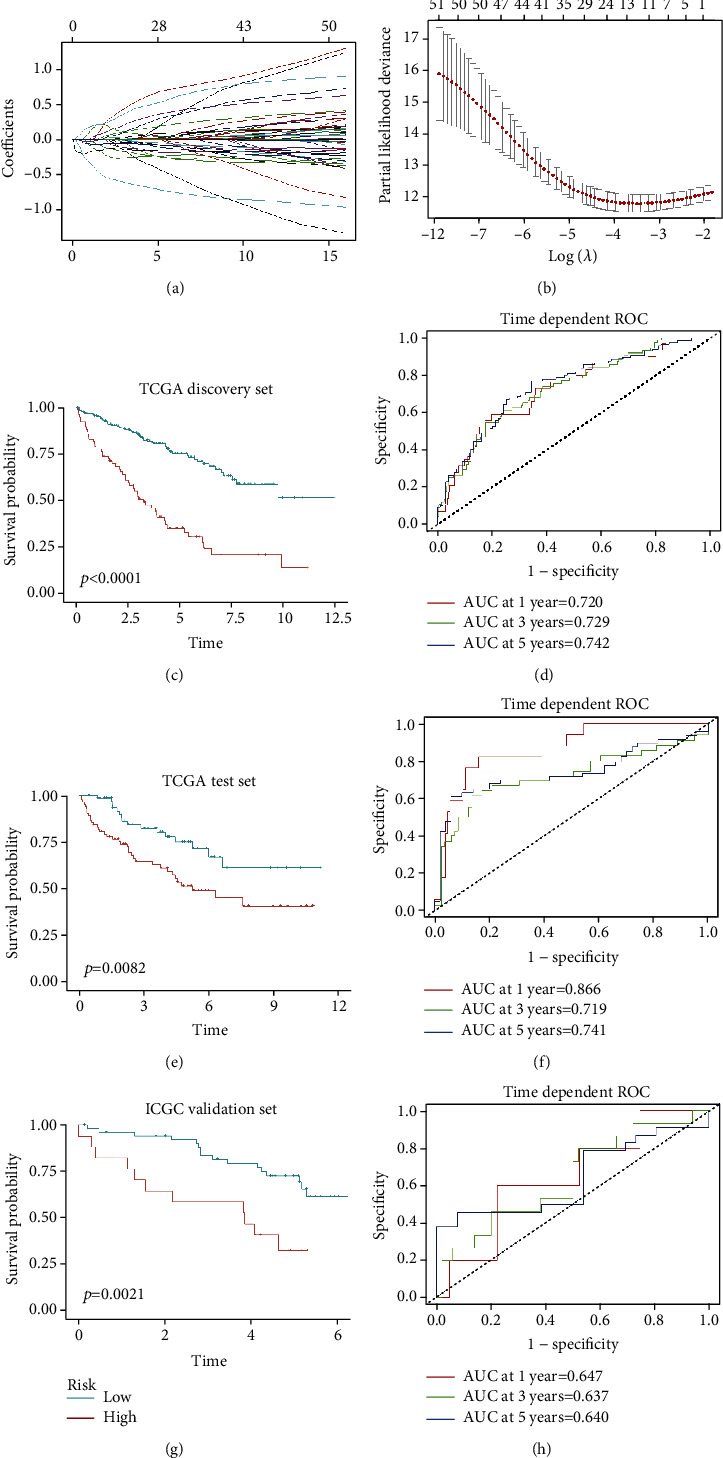
Construction and evaluation of the 12-gene risk model in KIRC. (a) The LASSO coefficient profiles of the 54 immune-related prognostic genes. (b) Partial likelihood deviance for LASSO coefficient profiles and optimal lambda selection in the LASSO model. (c, e) Kaplan-Meier analysis of TCGA KIRC discovery cohort and test cohort. (d, f) Time-dependent ROC curves displayed the predictive value of our prognostic model in TCGA discovery and test cohorts. (g) Kaplan-Meier analysis of ICGC external validation cohort. (h) Time-dependent ROC curves displayed the predictive value of our model in ICGC external validation dataset.

To further screen the prognostic genes from these two immune-related modules, we performed univariate Cox analysis of these 299 genes. Genes with *P* values of less than 0.001 were considered candidates to build the prognostic model. We obtained 54 genes that were correlated with KIRC outcomes. We then used TCGA discovery cohort containing 349 patient data as the training dataset and applied LASSO Cox regression analysis to identify stable markers from these 54 prognostic genes. 12 genes (*CAPZA1*, *EMX2*, *FGL2*, *FUCA1*, *GRB2*, *HLA-E*, *IMPA2*, *NFE2L3*, *PLEKHO1*, *RORC*, *SIGLEC1*, and *UBE2Z*) were included in the prognostic model (Figures 4(a) and 4(b)). Survival and ROC curve analyses were used to investigate the predictive accuracy of our risk model. In TCGA discovery cohort, we calculated the risk score based on the prognostic model and divided patients into two groups (high risk versus low risk); the optimal cutoff value was -4.192, which was based on the 1-year ROC curve. We then calculated the risk score of each patient, the high-risk score indicated shorter OS (Figure 4(c)). The areas under the ROC curves for 1-, 3-, and 5-year OS were 0.720, 0.729, and 0.742, respectively (Figure 4(d)). Next, we used TCGA test cohort and external datasets from the ICGC database evaluate the predictive value of our prognostic model. In TCGA test cohort, patients with lower risk score had longer OS (Figure 4(e)), and the areas under the ROC curves of this model in the test cohorts for 1-, 3-, and 5-year OS were 0.866, 0.719, and 0.741, respectively (Figure 4(f)). In the ICGC validation cohort, based on the optimal cutoff -3.276, risk score was closely correlated with patient survival (Figure 4(g)), and the areas under the ROC curves of this model in the ICGC validation datasets for 1-,3-, and 5-year OS were 0.647, 0.637, and 0.640, respectively (Figure 4(h)). Taken together, our prognostic model had good performance in predicting the outcome of patients with KIRC. Parameters for building the LASSO Cox model are shown in Table 2.

### 3.5. Twelve Genes in the Prognostic Model Correlated with Patient Outcomes

**Figure 5:**
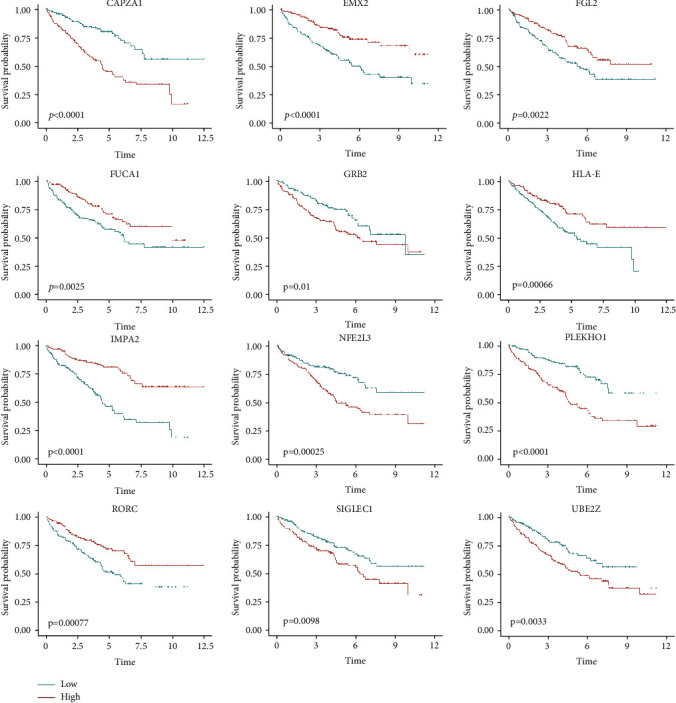
Survival analysis for the 12 genes in the prognostic model in TCGA KIRC cohort (log-rank test).

Next, we explored the correlations of these 12 genes in our prognostic model with patient outcomes. Based on the expression of each gene, we divided patients with KIRC into high-expression (expression > 67 percentile) and low-expression (expression ≤ 33 percentile) groups and plotted Kaplan-Meier curves. The expression levels of these 12 genes in our model significantly and independently influenced patient survival (log-rank test, *P* value < 0.05). Patients with high expression levels of *CAPZA1*, *GRB2*, *NF2EL3*, *PLEKHO1*, *SIGLEC1*, and *UBE2Z* and low expression levels of *FGL2*, *EMX2*, *FUCA1*, *HLA-E*, *IMPA2*, and *RORC* had poor prognosis (Figures 5(a)–5(l)).

### 3.6. Twelve-Gene Expression Signature

**Figure 6:**
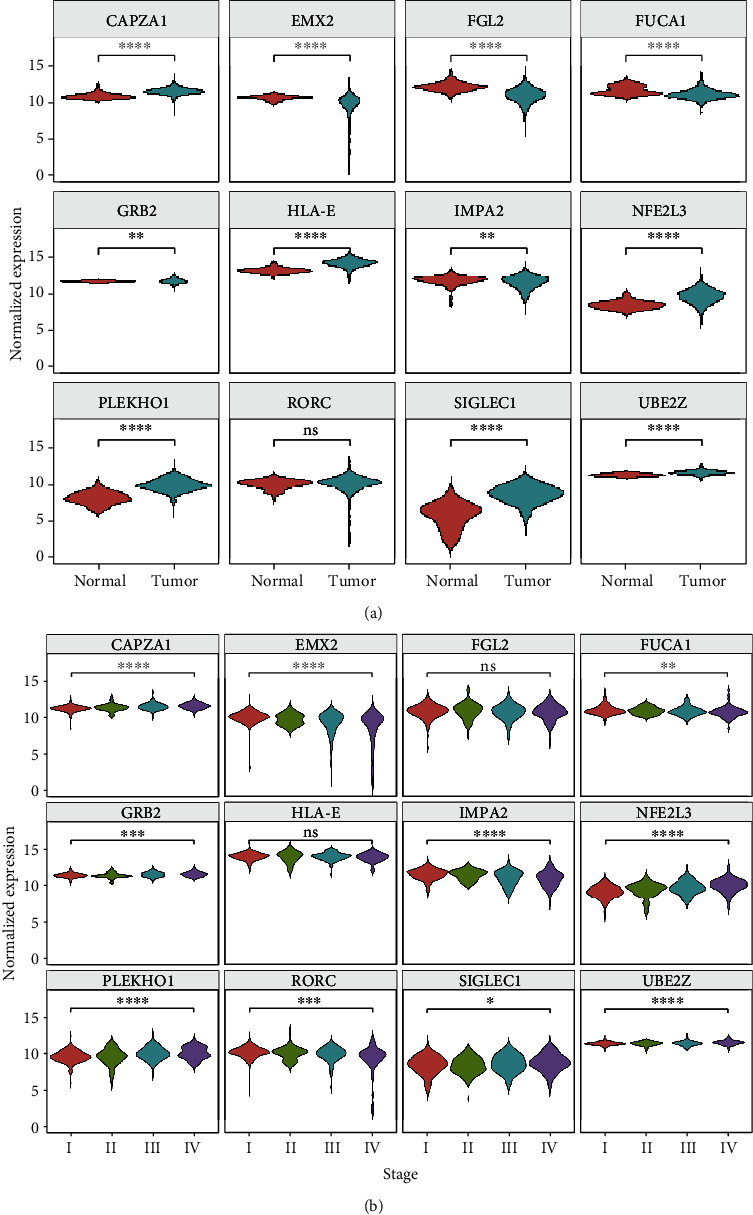
Analysis of expression of the 12 hub genes during KIRC tumorigenesis and progression. In TCGA dataset, expression of the 12 hub genes was evaluated (a) in tumor and normal tissues and (b) at different stages (*t*-test; ns: not significant; ^∗^*P* < 0.05,  ^∗∗^*P* < 0.01,  ^∗∗∗^*P* < 0.001, and^∗∗∗∗^*P* < 0.0001).

To investigate the dynamitic changes in the expression levels of these 12 genes during tumorigenesis and tumor progression, we compared their expression levels in tumor tissues and adjacent normal tissues. The expression levels of all genes, except *RORC*, showed significant differences among tumor and normal tissues. *CAPZA1*, *HLA-E*, *IMPA2*, *NFE2L3*, *PLEKHO1*, *SIGLEC1*, and *UBE2Z* were expressed at higher levels in tumor tissues, whereas *EMX2*, *FGL2*, *FUCA1*, and *GRB2* were expressed at lower levels in tumor tissues (Figure 6(a)). During tumor progression, *CAPZA1*, *GRB2*, *NF2EL3*, *PLEKHO1*, *SIGLEC1*, and *UBE2Z* were expressed at higher levels in late-stage KIRC, whereas *EMX2*, *FUCA1*, *IMPA2*, and *RORC* were expressed at higher levels in early-stage KIRC (Figure 6(b)).

### 3.7. Genes in the Prognostic Model Correlated with Immune Infiltration in KIRC

**Figure 7:**
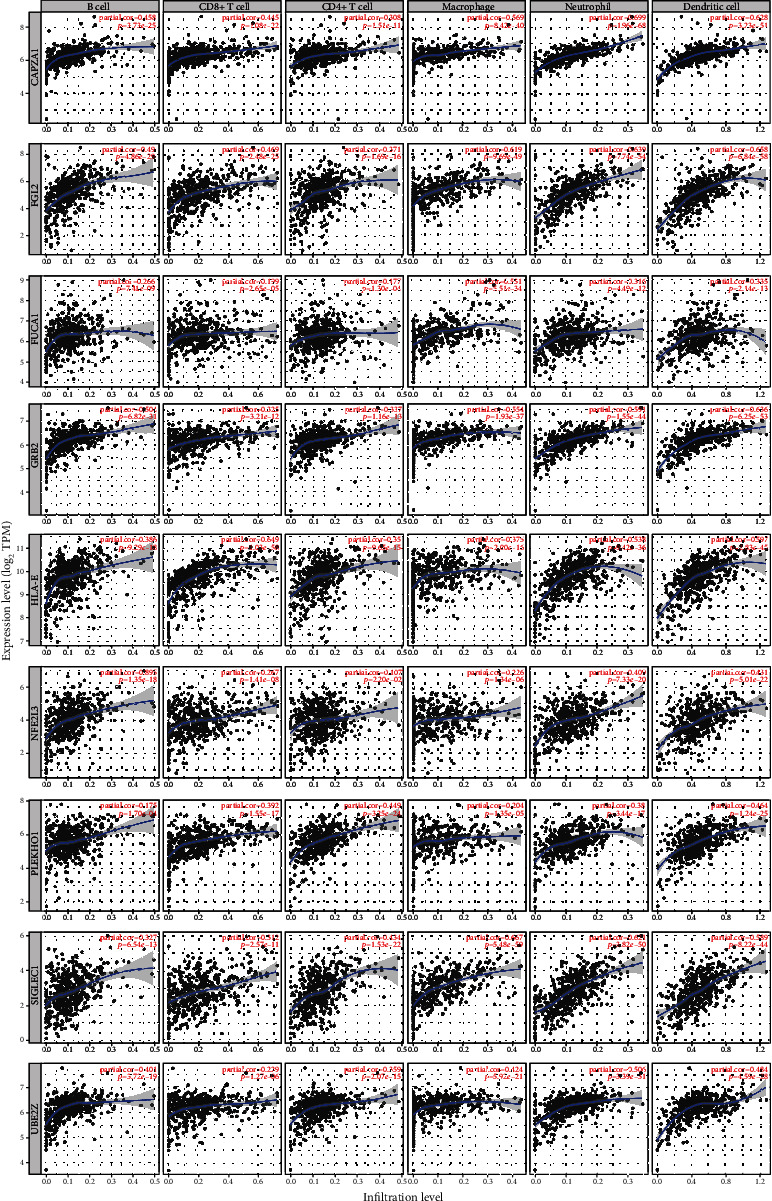
Correlations between model genes and infiltration levels of immune cells. Nine genes, including *CAPZA1*, *FGL2*, *FUCA1*, *GRB2*, *HLA-E*, *NFE2L3*, *PLEKHO1*, *SIGLEC1*, and *UBE2Z*, were closely associated with infiltration of immune cells in tumor.

Because our prognostic model was based on 12 genes that were closely correlated with immune scores, immune infiltration profiling was used to explore the influence of these genes on tumor-infiltrating immune cells within the TME. The proportions of immune cell types, including B cells, CD8^+^ T cells, CD4^+^ T cells, macrophages, neutrophils, and dendritic cells, in tumor samples were calculated by TIMER. The results showed that nine genes, including *CAPZA1*, *FGL2*, *FUCA1*, *GRB2*, *HLA-E*, *NFE2L3*, *PLEKHO1*, *SIGLEC1*, and *UBE2Z*, had significantly positive correlations with immune cell infiltration (*P* < 0.05, partial correlation > 0.3) (Figure 7).

### 3.8. Differences in Immune Cell Subtypes between the High- and Low-Risk Groups

**Figure 8:**
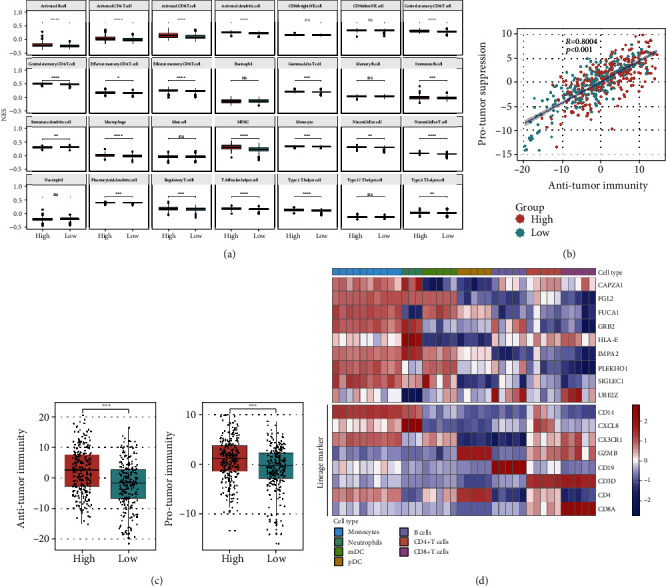
Immune cell infiltration heterogeneity in KIRC. (a) The proportions of 28 immune cells in KIRC were analyzed by ssGSEA. (b) Correlation between infiltration of antitumor immune cells and protumor immune cells. *R* coefficient of Pearson's correlation. The shaded area represents the 95% confident interval (*R* = 0.8004, *P* < 0.001). (c) Boxplot showing the relationship of antitumor immune cells and protumor immune cells in high- and low-risk groups (*t*-test; ns: not significant; ^∗^*P* < 0.05,  ^∗∗^*P* < 0.01,  ^∗∗∗^*P* < 0.001,  ^∗∗∗∗^*P* < 0.0001). (d) Heatmap of hub gene expression level in different immune cell subsets.

To explore the immune cell profiles within the KIRC microenvironment, the proportions of 28 immune cell types in KIRC were analyzed by ssGSEA (Figure 8(a)). Patients with high immune scores, which were correlated with high infiltration of immune cells in tumors, had poor outcomes. We further analyzed the regulatory mechanism of KIRC. Pearson's correlation analysis showed that the abundances of antitumor immune cells and protumor immune cells were positively associated within the TME (*R* = 0.8004, *P* < 0.001; Figure 8(b)). The proportions of antitumor immune cells and protumor immune cells were both significantly higher in the high-risk group (Figure 8(c)). Further, we analyzed the expression level of these hub genes in different immune cell subtypes. *EMX2*, *NFE2L3*, *RORC* failed to map to the probe on GeneChips. The expression levels of *CAPZA1*, *FGL2*, *FUCA1*, *GRB2*, *IMPA2*, *PLEKHO1*, and *SIGLEC1* were relatively higher in monocytes. The expression of *HLA-E* and *UBE2Z* was relatively abundant in NK cells and lymphocytes (Figure 8(d)). Taken together, the hub genes in our model were closely associated with the infiltration level and the composition of immune cells in the tumor microenvironment of KIRC.

## 4. Discussion

ccRCC is the most common subtype of kidney cancer. With improvements in our understanding of the molecular mechanisms (e.g., the VHL/hypoxia-inducible factor (HIF)/vascular endothelial growth factor pathway) of the tumorigenesis and progression of ccRCC, several targeted therapies have been applied in treatment [19]. Recently, several studies have revealed that the TME plays important roles in tumor malignancy, providing good opportunities to use immunotherapy in the management of patients with KIRC [8]. In our study, based on RNA-seq data from tumor tissues, we found that immune scores increased during tumor progression and were significantly associated with patient outcomes. Immune scores were significantly higher in tumor tissues from patients with advanced-stage cancer. However, differences in stromal scores were not significant at different stages of KIRC. Our results highlighted the importance of the immune microenvironment during the progression of KIRC. Consistent with a previous study [20], we found that patients with high immune scores typically had poor outcomes.

The composition and function of tumor-infiltrating immune cells affect tumor development through synergy or opposing effects. The immune system can be activated to kill tumor cells by exerting antitumor effects, whereas tumor-infiltrating immune cells can be inhibited to promote tumor progression and metastasis. Thus, the immune score may be a superior indicator to predict patient outcomes. In this study, we used WGCNA to identify gene modules that were closely associated with tumor stage, grade, immune score, and estimate score. GO and KEGG analyses of immune-related module genes revealed that these genes were related to adaptive and innate immune responses. Univariate Cox and LASSO Cox analysis were performed to identify the hub genes in the immune-related module and to construct a prognostic model. Finally, 12 genes were identified, and each of these genes could independently influence clinical outcomes. We used TCGA discovery dataset to construct a risk model. Then, we used TCGA test cohort and ICGC RECA dataset to evaluate the accuracy of our model. Our model showed good performance in the discovery cohort and internal and external test dataset. In summary, our model may have a potential value for predicting outcomes in patients with KIRC.

There are several grouped variable selection methods including Elastic net, LASSO, and Net. Lasso is a regularization technique for performing linear regression and includes a penalty term that constrains the size of the estimated coefficients. Therefore, it resembles ridge regression. Lasso is a shrinkage estimator: it generates coefficient estimates that are biased to be small. In our research, Lasso is the most prevalent technique to attain the main features among a branch of features in small models.

In our study, patients with KIRC with high expression levels of *CAPZA1*, *GRB2*, *NFE2L3*, *PLEKHO1*, *SIGLEC1*, and *UBE2Z* had poor outcomes, whereas those with high expression levels of *EMX2*, *FGL2*, *FUCA1*, *HLA-E*, *IMPA2*, and *RORC* had better outcomes. The expression levels of nine genes, including *CAPZA1*, *EMX2*, *FUCA1*, *GRB2*, *IMPA2*, *NFE2L3*, *PLEKHO1*, *SIGLEC1*, and *UBE2Z*, were significantly different during tumorigenesis, and their expression showed gradually changes with the progression of KIRC. *CAPZA1* is involved in the EMT and autophagy in tumors [21, 22], and we found monocytes have relatively high expression level of *CAPZA1*. In hepatocellular carcinoma, *CAPZA1* is also associated with the HIF-1*α* pathway, which is important during RCC tumorigenesis. Downregulation of the *EMX2* gene participates in tumor metastasis and reduced overall survival [23]. In our study, patients with low expression of *EMX2* had poor outcomes. *FUCA1*, encoding alpha-l-fucosidase 1, is a target of p53, and loss-of-function mutations in *FUCA1* are found in several cancers. *GRB2* is associated with intracellular signal transduction. *GRB2* signaling is essential for the cell cycle, cell motility, angiogenesis, and vasculogenesis [24]. High expression of *GRB2* in patients with KIRC may be related to tumor metastasis and could indicate a poor prognosis. Studies have found that downregulation of *IMPA2* is associated with poor outcomes in ccRCC and that *miR-25*-mediated *IMPA2* regulation could be a potential therapeutic target [25]. In this study, we also showed that *IMPA2* was important in kidney tumors. Moreover, *NFE2L3* may influence ccRCC progression by regulating immune activity, including antigen presentation and the NOD-like receptor signaling pathway [26]. *PLEKHO1* affects tumor cell proliferation and apoptosis. Downregulation of *PLEKHO1* impairs RCC progression [27]. In our study, *PLEKHO1* was found to be highly expressed in monocyte and DC. *PLEKHO1* might potentially affect the tumor immune microenvironment and could therefore be a novel target of immunotherapy. Several studies have suggested that *SIGLEC1*, which encodes CD169, can act as a tumor-associated macrophage biomarker [28, 29]. High density of macrophages in tumors is usually a negative prognostic marker. Our studies also showed that *SIGLEC1* was correlated with the levels of infiltration of macrophages in ccRCC and the expression level of *SIGLEC1* was higher in monocyte and DC. Patients with high *SIGLEC1* expression had a poor prognosis. *UBE2Z* encodes ubiquitin conjugating enzyme E2 Z, a member of the ubiquitin-conjugating enzyme family. In hepatocellular carcinoma, *UBE2Z* is overexpressed in tumor tissues and is significantly associated with TNM stage and histological grade [30]. In our study, a similar expression pattern was also observed, suggesting that *UBE2Z* may be a good prognostic indicator in KIRC.

*FGL2*, encoding fibrinogen-like protein 2, and *HLA-E*, encoding MHC class I antigen E, were significantly differentially expressed in tumor tissues; however, their expression levels were comparable at different stages of ccRCC. We found mononuclear phagocytic cells had higher expression level of *FGL2* among different immune cell subtypes. *FGL2* can modulate immune reactions and may be a potential immunotherapeutic target in glioma [31]. *FGL2* is significantly correlated with infiltrating levels of immune cells in tumors and may be a potential therapeutic target in KIRC. Studies in colorectal cancer have shown that *HLA-E* is correlated with tumor metastasis and has a predictive value for OS [32]. *HLA-E* is a ligand for the inhibitory CD94/NKG2A receptor and is reported to affect the functions of tumor-infiltrating CD8^+^ T cells [33]. NK cells and T lymphocytes expressed relatively high level of *HLA-E*. Our findings revealed that *HLA-E* was significantly correlated with infiltrating levels of CD8^+^ T cells in tumors. We also found that expression of *RORC* was comparable in tumor and normal tissues, although its expression level was significantly lower in advanced-stage tumor tissues. *RORC* is a regulator of the proinflammatory Th17/interleukin-17 axis in adult T-cell leukemia [34], and low expression of *RORC* was a negative prognostic indicator of KIRC in our study. Thus, further studies are needed to assess the potential functions and mechanisms of *RORC* in immunotherapy.

We used TIMER to analyze the correlations between the hub genes and the infiltration levels of immune cells. Nine genes were identified to be associated with infiltration of immune cells. And in the GEO dataset, we investigated the expression levels of hub genes in different immune cell subtypes. The results indicated that *CAPZA1*, *FGL2*, *FUCA1*, *GRB2*, and *SIGLEC1* were relatively high expressed in monocyte, and these genes had significantly positive associations with the infiltration level of macrophage in KIRC. *FGL2*, *FUCA1*, *PLEKHO1*, and *SIGLEC1* were highly expressed in DC and might influence its number in tumor. The levels of *HLA-E* and *UBE2Z* were higher in lymphocytes, and their expression levels were correlated with the infiltration levels of B cell and T cell in tumor tissue. The expression levels of these hub genes can indicate the abundance of immune cells in tumor tissue, and their function in KIRC immunity needs to be further investigated.

To further explore the correlations between immune cell infiltration and risk score, we used ssGSEA to calculate the proportions of immune cell subtypes in tumors. Our results showed that the abundances of various immune cell subtypes were different between the high- and low-risk score groups. Cells showing antitumor activity, such as increased levels of activated CD8^+^ T cells, Th1 cells, and activated dendritic cells, were significantly more abundant in the high-risk group. Moreover, cells such as MDSCs and regulatory T cells, which are suppressed by protumor responses, were also more abundant in the high-risk group. Pearson's correlation analysis showed that antitumor immunity and protumor suppression were significantly positively associated within the TME. Additionally, patients with KIRC with higher immune scores typically have poor clinical outcomes and higher immune scores correlated with higher infiltration of immune cells in tumors. The antitumor immunity and protumor suppression scores in high-risk patients were both higher than those in the low-risk group. This phenomenon suggested that there may be a feedback mechanism for immune suppression in patients with KIRC.

**Figure 9:**
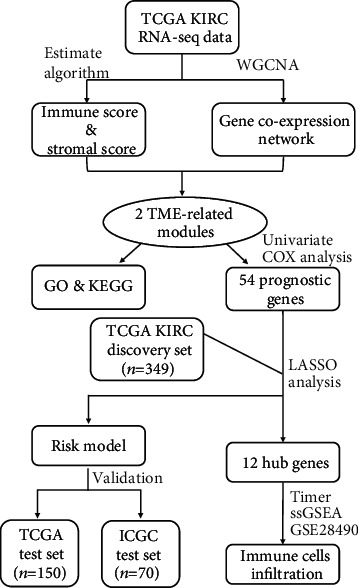
Workflow of current work.

In our study, we constructed a prognostic model of KIRC based on TCGA cohort. Next, we used the internal validation dataset from TCGA and the external validation dataset from ICGC to evaluate our model. Furthermore, we used various algorithms and GEO datasets to explore the relationship between hub genes and tumor microenvironment (Figure 9). However, our study had some limitations. First, our prognostic model needs to be validated using prospective clinical studies. Second, the underlying mechanism should be further confirmed by experiments.

## 5. Conclusions

This study identified 12 hub genes that were closely associated with the tumor immune microenvironment in patients with KIRC and constructed a prognostic model to predict patient outcomes. Nine genes, including *CAPZA1*, *FGL2*, *FUCA1*, *GRB2*, *HLA-E*, *NFE2L3*, *PLEKHO1*, *SIGLEC1*, and *UBE2Z*, were significantly associated with infiltration levels of immune cells, indicating they may be therapeutic targets of KIRC.

## Figures and Tables

**Table 1:** Clinical characteristics of KIRC in TCGA and ICGC datasets.

KIRC patient clinical characteristics			
	TCGA dataset (*N* = 499)		ICGC dataset (*N* = 70)
Age	Discovery set (*N* = 349)	Test set (*N* = 150)	
<60	172 (49.3%)	63 (42.0%)	32 (45.7%)
≥60	177 (50.7%)	87 (58.0%)	38 (54.3%)
Gender			
Female	110 (31.5%)	62 (41.3%)	29 (41.4%)
Male	239 (68.5%)	88 (58.7%)	41 (58.6%)
Vital status			
Deceased	113 (32.4%)	53 (35.3%)	27 (38.6%)
Living	236 (67.6%)	97 (64.7%)	43 (61.4%)
Histologic grade			
G1	7 (2.0%)	5 (3.3%)	NA
G2	155 (44.4%)	61 (40.7%)	NA
G3	138 (39.5%)	60 (40.0%)	NA
G4	46 (13.2%)	23 (15.3%)	NA
GX	3 (0.9%)	1 (0.7%)	NA
Stage			
I	170 (48.7%)	81 (54.0%)	NA
II	39 (11.2%)	12 (8.0%)	NA
III	87 (24.9%)	29 (19.3%)	NA
IV	53 (15.2%)	28 (18.7%)	NA
T classification			
T1	175 (50.1%)	82 (54.7%)	37 (52.9%)
T2	47 (13.5%)	16 (10.7%)	13 (18.6%)
T3	123 (35.2%)	47 (31.3%)	19 (27.1%)
T4	4 (1.1%)	5 (3.3%)	1 (1.4%)
N classification			
N0	154 (44.1%)	70 (46.7%)	60 (85.7%)
N1	10 (2.9%)	5 (3.3%)	2 (2.9%)
NX	185 (53.0%)	75 (50.0%)	8 (11.4%)
M classification			
M0	285 (81.7%)	115 (76.7%)	61 (87.1%)
M1	50 (14.3%)	26 (17.3%)	8 (11.4%)
MX	14 (4.0%)	9 (6.0%)	1 (1.4%)

**Table 2:** Twelve-gene prognostic model by LASSO regression in the KIRC discovery cohort.

Gene	Coef	HR	HR.CI	Cox *P* value
*CAPZA1*	0.206861	2.63	1.88~3.67	1.69*E* − 08
*EMX2*	-0.00295	0.80	0.75~0.86	1.49*E* − 10
*FGL2*	-0.10874	0.79	0.69~0.90	0.000457
*FUCA1*	-0.51746	0.56	0.43~0.75	5.41*E* − 05
*GRB2*	0.196888	1.37	1.15~1.63	0.000475
*HLA-E*	-0.20349	0.66	0.52~0.84	0.000681
*IMPA2*	-0.14589	0.60	0.52~0.70	2.15*E* − 11
*NFE2L3*	0.038322	1.54	1.3~1.82	3.38*E* − 07
*PLEKHO1*	0.215124	1.61	1.35~1.93	1.46*E* − 07
*RORC*	-0.06741	0.71	0.63~0.79	1.37*E* − 09
*SIGLEC1*	0.093055	1.24	1.09~1.40	0.000896
*UBE2Z*	0.008479	2.78	1.65~4.67	0.000124

## Data Availability

Publicly available datasets were analyzed in this study. This data can be found in the following: https://xenabrowser.net/datapages/?cohort=GDC%20TCGA%20Kidney%20Clear%20Cell%20Carcinoma%20(KIRC)~~~~~~~~~^~^~^~^~~~~~~~~~~~amp; http://2Fxena.treehouse.gi.ucsc.edu; https://gdac.broadinstitute.org/; https://dcc.icgc.org/releases/current/Projects/RECA-EU; and https://www.ncbi.nlm.nih.gov/geo/query/acc.cgi?acc=GSE28490.
